# IGF2BP2 promotes cell invasion and epithelial-mesenchymal transition through Src-mediated upregulation of EREG in oral cancer

**DOI:** 10.7150/ijbs.91786

**Published:** 2024-01-01

**Authors:** Chiao-Wen Lin, Wei-En Yang, Chun-Wen Su, Hsueh-Ju Lu, Shih-Chi Su, Shun-Fa Yang

**Affiliations:** 1 Institute of Oral Sciences, Chung Shan Medical University, Taichung, Taiwan.; 2 Department of Dentistry, Chung Shan Medical University Hospital, Taichung, Taiwan.; 3 Institute of Medicine, Chung Shan Medical University, Taichung, Taiwan.; 4 Department of Medical Research, Chung Shan Medical University Hospital, Taichung, Taiwan.; 5 Division of Hematology and Oncology, Department of Internal Medicine, Chung Shan Medical University Hospital, Taichung, Taiwan.; 6 School of Medicine, Chung Shan Medical University, Taichung, Taiwan.; 7 Whole-Genome Research Core Laboratory of Human Diseases, Chang Gung Memorial Hospital, Keelung, Taiwan.; 8 Department of Medical Biotechnology and Laboratory Science, College of Medicine, Chang Gung University, Taoyuan, Taiwan.

**Keywords:** insulin-like growth factor 2 mRNA binding protein 2, oral squamous cell carcinoma, epithelia-mesenchymal transition

## Abstract

Insulin-like growth factor 2 mRNA binding protein 2 (IGF2BP2), with high affinity to a myriad of RNA transcripts, has been shown to elicit promotive effects on tumorigenesis and metastasis. Yet, the functional involvement of IGF2BP2 in the progression of oral squamous cell carcinoma (OSCC) remains poorly understood. In this study, we showed that IGF2BP2 was upregulated in head and neck cancer, and high levels of IGF2BP2 were associated with poor survival. In *in vitro* experiments, IGF2BP2 promoted migration and invasion responses of OSCC cells. Moreover, we identified an IGF2BP2-regulated gene, EREG, which functioned as a modulator of OSCC invasion downstream of IGF2BP2. In addition, EREG expression triggered the epithelia-mesenchymal transition (EMT) in OSCC, as evidenced by the observation that knockdown of EREG weakened the induction of EMT mediated by IFG2BP2, and replenishment of EREG favored the EMT in IGF2BP2-depleted cells. Such IGF2BP2-regulated EREG expression, EMT, and cell invasion were dependent on the activation of FAK/Src signaling pathway. Collectively, these findings suggest that EREG, serving as a functional mediator of IGF2BP2-regulated EMT and cell invasion in oral cancer, may be implicated as a potential target for antimetastatic therapies.

## Introduction

Oral squamous cell carcinoma (OSCC), accounting for a vast majority of oral cancer 1, is a common type of malignancies globally and especially prevalent in Southeast Asia 2. Despite current advancements in treatment options, the mortality of patients with oral cancer has remained mostly unchanged over past decades 3, largely because of tumor metastasis. OSCC dissemination, is a sophisticated process during which tumor cells are highly invasive and prone to escape from the original location and propagate at a secondary site 4-6. This cancer hallmark is known to be orchestrated by the actions of many cell-intrinsic identities through various genetic factors and extrinsic parameters 7, 8. Improvements on the understanding of oral cancer dissemination are crucial for dissecting the molecular mechanisms of OSCC progression but also for the developing novel therapeutic interventions.

Insulin-like growth factor 2 (IGF2) mRNA binding protein 2 (IGF2BP2) was originally identified as a binding protein of IGF2 mRNA 9 and known to regulate the localization, stability, and translation of RNA molecules 10. Through a post-transcriptional regulation of several genes in various cell types, a key role of IGF2BP2 in cellular metabolism and impaired insulin secretion has been demonstrated 11. In addition to binding of many mRNA targets, IGF2BP2 recently was shown to interact with N6-methyladenosine-modified long noncoding RNAs 12. Based on its wild spectrum of interacting RNA partners, IGF2BP2 has been functionally involved in a variety of biological and pathogenic processes, including the development and progression of numerous malignancies 13-15. In many cancer types, upregulation of IGF2BP2 has been associated with poor disease prognosis 16-18, implicating IGF2BP2 as a tumor promoter. Mechanistically, IGF2BP2 was shown to govern cancer metabolism, apoptosis, invasion, and metastasis by regulating different types of RNA species post-transcriptionally in numerous cell types and pathways 19. Recently, an intriguing study reported that IGF2BP2 promoted the degradation of the RNA transcript encoding ATP6V1A, a catalytic subunit of the vacuolar ATPase, thus impairing lysosomal function and resulting in a unique secretome that greatly enhances breast cancer cell invasiveness 20. Yet, as N6-methyladenosine modification of various RNA species has been implicated in regulating many aspects of OSCC biology 21-23, the involvement of IGF2BP2 in influencing oral cancer progression remains largely unclear. Here, through conducting a transcriptome analysis, we demonstrated that expression of epiregulin (encoded by the EREG gene) was positively regulated by IGF2BP2, accompanied by increases in levels of several epithelial-mesenchymal transition genes and oral cancer migration. Our results provide extra insight into the mechanisms underlying the promotive role of IGF2BP2 in OSCC invasion.

## Materials and Methods

### Cell lines and reagents

Human OSCC cell lines, CAL-27, HSC-3, and SCC-9 cells were obtained from the American Type Culture Collection (ATCC, Manassas, VA, USA). Other OSCC cells, Ca9-22, SAS, and SCC-14 cells were purchased from the Japanese Collection of Research (JCRB, Osaka, Japan). Human oral keratinocytes (HOK) were gained from ScienCell Research Laboratories (Carlsbad, CA, USA). OSCC cell lines were propagated in Dulbecco's modified Eagle's medium (MEM, Life Technologies, Grand Island, NY, USA) containing 10% fetal bovine serum. HOK cells were maintained in oral keratinocyte medium (ScienCell), and SG cells were cultured in MEM-F12 medium (Life Technologies). All cell cultures were kept at 37 °C in a humidified atmosphere of 5% CO2. PP1 (a Src inhibitor) of HPLC grade with ≥98% purity was obtained from Sigma-Aldrich and dissolved in DMSO.

### Immunoblotting

Cell lysates were collected and subjected to SDS-PAGE analyses 24. Primary antibodies targeting the following proteins were used for detection: Anti-IGF2BP2 (ab124930), Anti-EREG (ab233512), Anti-E-cadherin (ab238099), Anti-FAK (ab40794) antibodies from Abcam (Cambridge, UK); Anti-fibronectin (#26836), Anti-vimentin (#5741), Anti-ZO-1 (#5406), Anti-Phospho-FAK (#3283), Anti-c-Raf (#9422), Anti-Phospho-c-Raf (#9427), Anti-Src (#2108), Anti-Phospho-Src (#2101), Anti-MEK1/2 (#9122), and Anti-Phospho-MEK1/2 (#9121) antibodies from Cell Signaling Technology (Danvers, MA, USA); Anti-β-actin (sc-47778) from Santa Cruz (Dallas, TX, USA); Horseradish peroxidase-conjugated secondary antibodies (Dako Corporation, Carpinteria, CA, USA). Densitometry results were processed by ImageJ software.

### Real-time RT-PCR

Briefly, total RNA was isolated using Trizol reagent, and first-strand cDNA was synthesized by using the High Capacity cDNA Reverse Transcription Kit. PCR amplification was carried out as described previously 25. Gene-specific primers were designed by Primer3 software, and levels of expression were evaluated by using the comparative Ct method.

### Gene silencing and expression

For silencing of IGF2BP2 or EREG, transfection of siRNAs (small interfering RNA) designed by the BLOCK-iT^TM^ RNAi Designer (ThermoFisher) was conducted. HSC-3 or SCC-14 cells were transfected with control or specific siRNAs (20-50 nM) against IGF2BP2 or EREG using the Lipofectamine 2000 transfection reagent (Life Technologies). The efficiency of gene knockdown was evaluated by using real-time PCR, and cells were used for subsequent experiments 48 hr after transfection. For overexpression, the cDNA encoding full-length IGF2BP2 and EREG was amplified by PCR and subcloned in the pENTER (Invitrogen) and pCMV6 (OriGene), respectively. Indicated expression plasmids and empty vector (as a control) were transfected into OSCC cells for gene expression.

### Flow cytometry

Cell cycle of OSCC cells with silencing or overexpression of IGF2BP2 was examined by accessing levels of cellular DNA with flow cytometry as described previously 26. In brief, cells were transfected with indicated plasmids for 48 hr and stained with PI (Invitrogen, Carlsbad, CA, USA), and the distribution of cell cycle was evaluated by a BD AccuriTM C6 Plus personal flow cytometer (BD Biosciences, San Jose, CA, USA).

### Cell migration and invasion assay

A modified Boyden chamber assay without and with 10 μL of Matrigel (25 mg/50 mL; BD Biosciences, MA) coating was used to assess cell migration and invasion, respectively, as described previously 27. In brief, cells were transfected with indicated plasmids for 48 hr and then seeded on the 8-μm-pore size polycarbonate membrane filter at 10^4^ cells/well in serum-free media. Cells were allowed to migrate or invade for 24 hr and counted under an Olympus CKX41 microscope (Olympus Corporation, Tokyo, Japan).

### RNA sequencing and data analysis

Total RNAs from HSC-3 cells with or without silencing of IGF2BP2 were isolated by TRIzol reagent (Invitrogen). Removal of ribosomal RNA, cDNA synthesis, and library construction and purification were performed as described previously 27. High-throughput sequencing was conducted on an Illumina Hiseq 2000 platform and paired-end reads of 100 bp were generated following manufacturer's protocol. Approximately, 3 gigabases of sequencing data per sample were produced. QC of RNA sequence reads was carried out before reads were aligned by TopHat2 28 and then reconstructed with Cufflinks 29 and Scripture 30. The gene or exon level expression was normalized to the number of reads per kilobase per million mapped reads (RPKM) 31. Genes with a *p* value smaller than 0.05 and a log2 value larger than 1 are considered as differentially expressed genes.

### Statistical Analysis

For all quantitative analyses, data are shown as mean ± standard deviation (SD) from at least three separate experiments. The threshold of difference was set by a *p* value of <0.05 using Student's t-test. The differences of gene expression levels in distinct tumor groups from The Cancer Genome Atlas (TCGA) were compared by the Mann-Whitney U test. The survival of patients was estimated with a Kaplan-Meier plotter and compared by using the log-rank test. The association of IGF2BP2 and EREG expression were analyzed by Pearson correlation from the TCGA and GSE84846 dataset.

## Results

### Clinical relevance of IGF2BP2 levels in head and neck tumor

**Figure 1:**
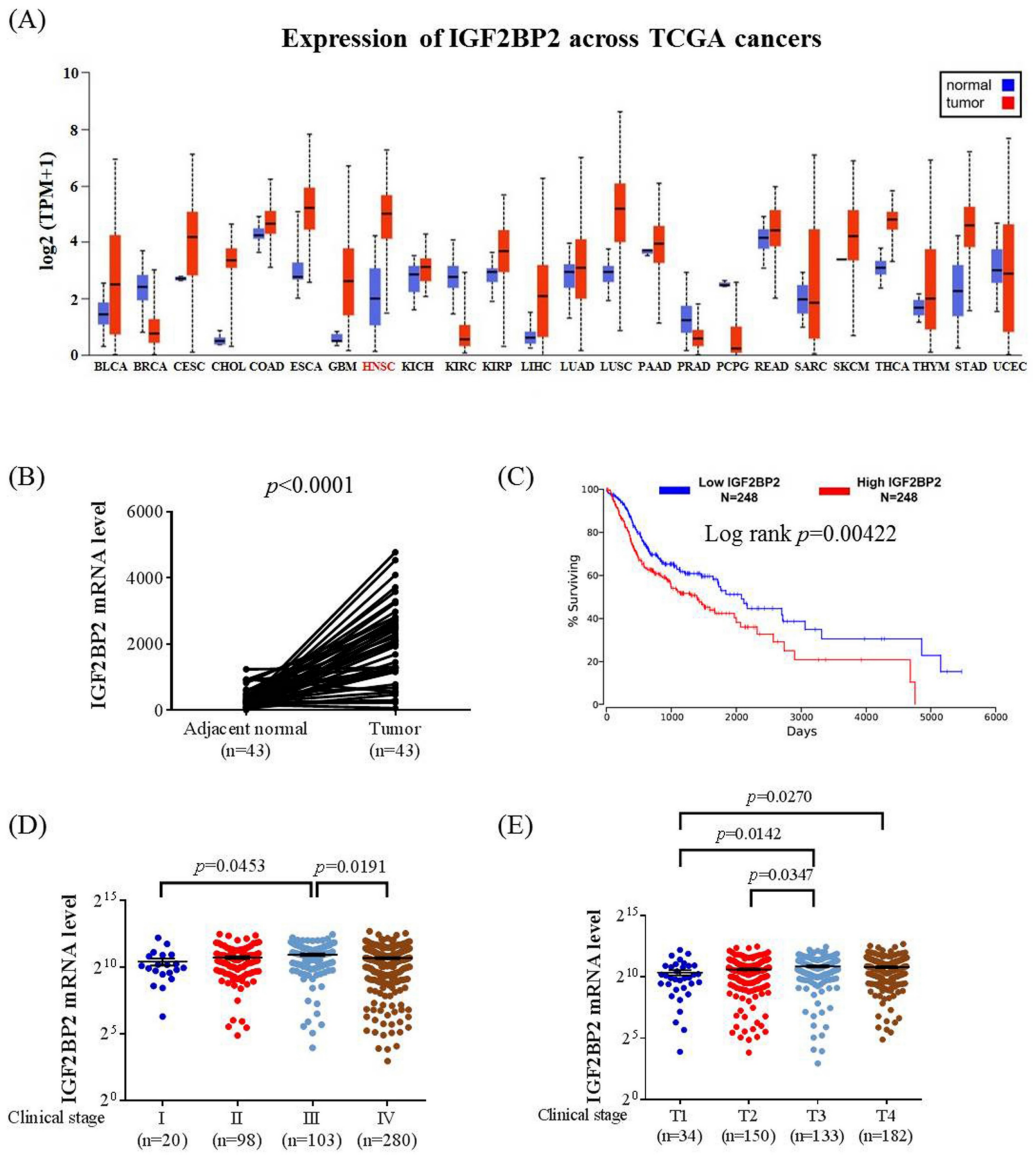
** IGF2BP2 expression levels are increased and associated with clinicopathological parameters in head and neck squamous cell carcinoma (HNSC). (A)** Comparison of IGF2BP2 expression between tumor and normal tissues across different cancer types from The Cancer Genome Atlas (TCGA) database.** (B)** Comparison of IGF2BP2 expression between HNSC specimens and their paired adjacent normal counterparts. **(C)** Survival analysis of patients with HNSC based on IGF2BP2 expression. *p* value was analyzed by log-rank test.** (D-E)** Correlations of IGF2BP2 expression with the clinical staging** (D)** and tumor size** (E)** of HNSC from The Cancer Genome Atlas (TCGA) database. The total number of samples is given in brackets. Mann-Whitney U test was used to verify the correlation between IGFBP2 and clinical staging and tumor size of HNSC.

To explore clinical insights of IGF2BP2 expression in OSCC, we surveyed public datasets from The Cancer Genome Atlas (TCGA). We found that IGF2BP2 expression was fluctuated across different cancer types in comparison with their corresponding normal tissues (**Figure 1A**). Significant elevation of IGF2BP2 expression in patients with head and neck squamous cell carcinoma (HNSC) was observed, whereas its expression was decreased in some other cancers, such as breast cancer and renal clear cell carcinoma. Additional analysis of HNSC specimens with their paired adjacent normal counterparts further demonstrated IGF2BP2 upregulation in HNSC cancer (**Figure 1B**,). In addition, augmented IGF2BP2 expression was associated with decreased survival, advanced clinical stage, and larger tumor size in HNSC patients (**Figure 1C-E**). These data imply a connection between IGF2BP2 expression and HNSC progression.

### Expression levels of IGF2BP2 affect oral cancer cell invasion

**Figure 2:**
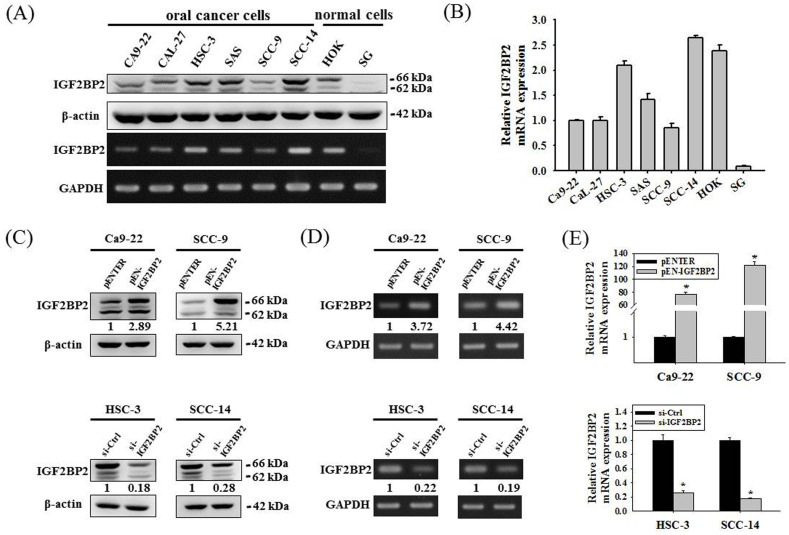

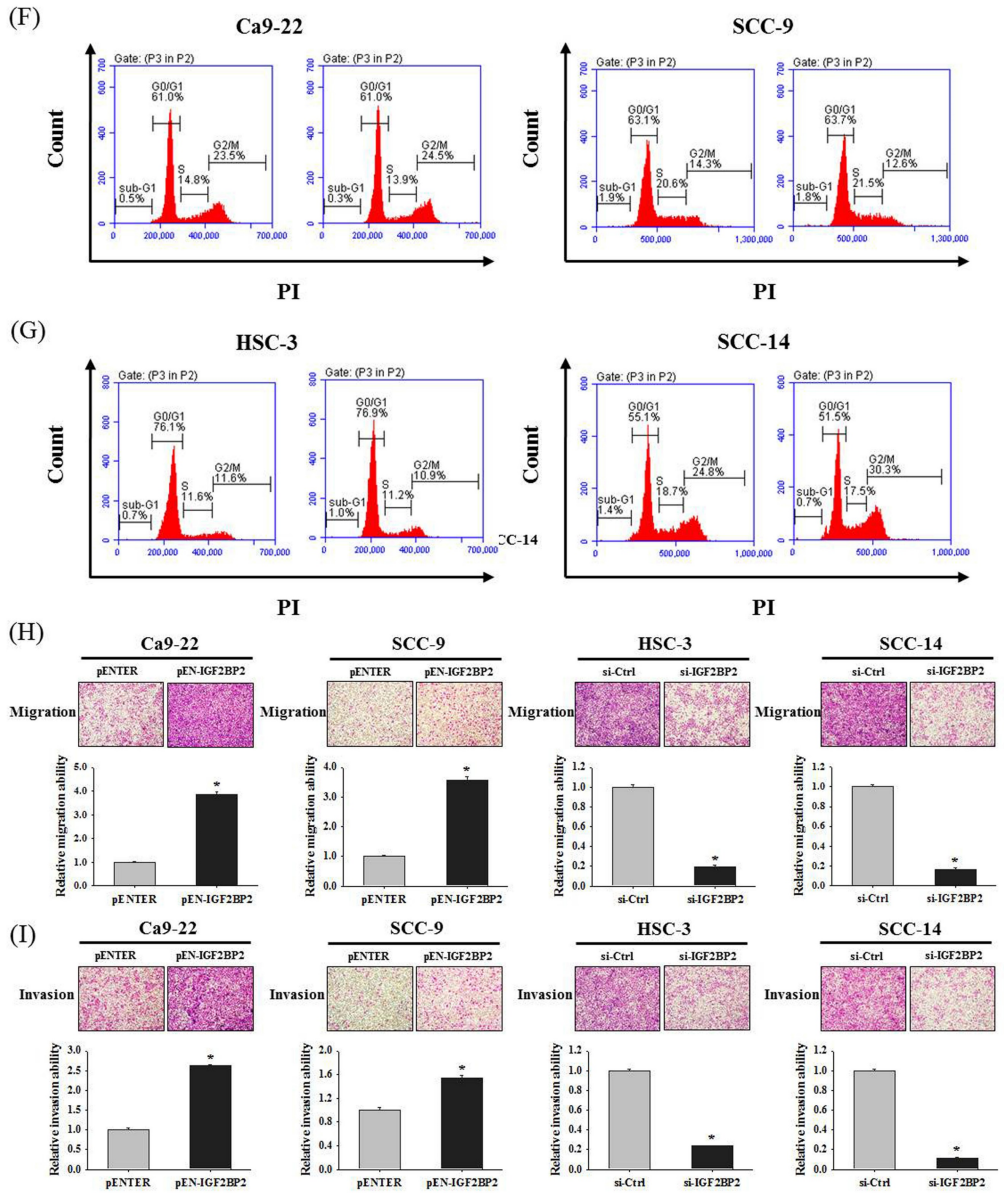
** IGF2BP2 promotes OSCC cell invasion.** (**A**) Basal levels of IGF2BP2 across OSCC cell lines and normal primary cells. Total RNA and protein lysate were collected from indicated cells and analyzed for the expression of IGF2BP2. (**B**) Relative expression levels of IGF2BP2 across OSCC cell lines and normal primary cells. Levels of IGF2BP2 RNA from individual cell lines were analyzed by real-time RT-PCR, normalized with the levels of GAPDH, and compared to Ca9-22 cells. **(C-E)** Verification of successful gene silencing and expression. OSCC cells transfected with indicated expression vectors or siRNAs were analyzed for their IGF2BP2 levels by immunoblotting **(C)**, RT-PCR **(D)**, and real-time RT-PCR **(E)**. Densitometric analyses were quantified by ImageJ and normalized with internal controls (β-actin and GAPDH). Data represent the mean ± SD of three independent experiments. *p < 0.05, compared with vehicle controls using Student's t-test. **(F-G)** DNA contents of OSCC cells with manipulation of IGF2BP2 levels were monitored by PI staining using flow cytometry. **(H-I)** OSCC cells with manipulation of IGF2BP2 levels were evaluated for their migration and invasion responses in a modified Boyden chamber without and with Matrigel coating, respectively. Quantitative results are shown underneath. *p < 0.05, compared with the vehicle control using Student's t-test.

We next examined the expression levels and potential functions of IGF2BP2 in OSCC. Our data showed that IGF2BP2 was expressed across a variety of OSCC cell lines (**Figure 2A-B**). In addition, IGF2BP2 expression was detectable in primary keratinocytes from human oral mucosa (HOK) but not in another normal oral epithelial cell line *(*SG). To explore the functional impact of IGF2BP2 on OSCC, we manipulated its levels by forced expression in Ca9-22 and SCC-9 cells and gene silencing in HSC-3 and SCC-14 cells, based on the endogenous expression levels of these cell lines (**Figure 2C-E**). We observed that changes in IGF2BP2 levels did not affect cancer cell cycle progression (**Figure 2F-G**) but consistently altered the migration and invasion responses in OSCC cells (**Figure 2H-I**). These findings reveal a functional role of IGF2BP2 in mediating OSCC invasion.

### IGF2BP2-regulated EREG expression is a regulator of OSCC invasion

**Figure 3:**
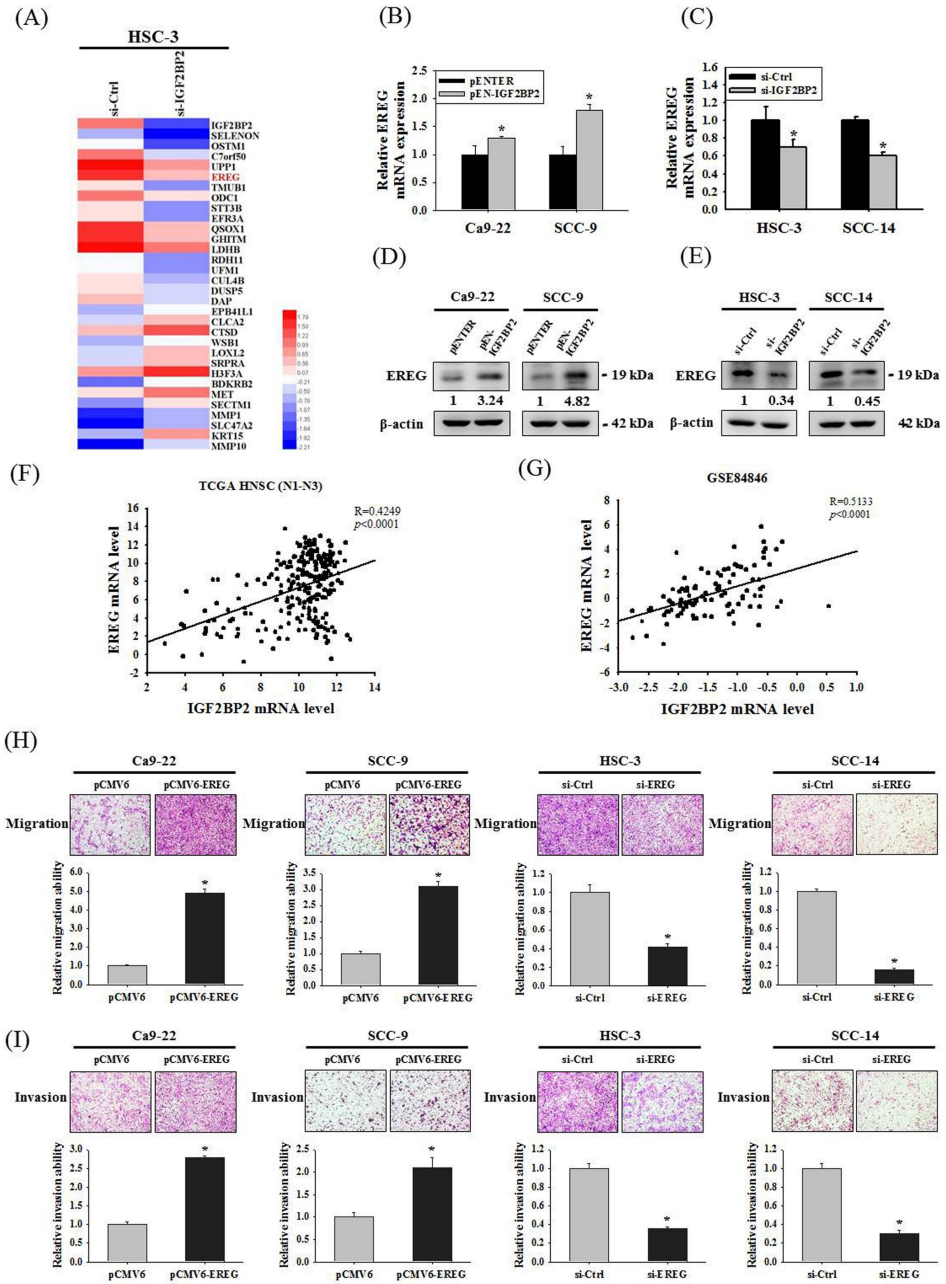
** Identification of EREG as an IGF2BP2-regulated gene that modulates OSCC invasion. (A)** Heatmap of the differentially expressed genes between HSC-3 cells with and without silencing of IGF2BP2. **(B-E)** OSCC cells transfected with indicated expression vectors or siRNAs were analyzed for their EREG levels by real-time RT-PCR **(B-C)** and immunoblotting** (D-E)**. *p < 0.05, compared with the vehicle control using Student's t-test. **(F-G)** Correlation of IGF2BP2 and EREG expression in clinical specimens of HNSC **(F)** and OSCC **(G)**. **(H-I)** OSCC cells with manipulation of EREG levels were evaluated for their migration and invasion responses in a modified Boyden chamber without and with Matrigel coating, respectively. Quantitative results are shown underneath. *p < 0.05, compared with the vehicle control using Student's t-test.

Since a functional impact of IGF2BP2 on OSCC invasion was noted, we next attempted to investigate the underlying molecular mechanisms through performing a transcriptome analysis. A list of genes were differentially expressed in HSC-3 cells as IGF2BP2 expression was reduced (**Figure 3A**). Among these genes, positive regulation of EREG expression by IGF2BP2 was consistently observed in OSCC cells (**Figure 3B-E**). EREG encodes a secreted protein, epiregulin, belonging to the epidermal growth factor (EGF) family of proteins. Specifically, forced expression of IGF2BP2 led to an elevation of EREG levels in CA-9-22 and SCC-9 cells, whereas knockdown of IGF2BP2 decreased EREG levels in HSC-3 and SCC-14 cells. Such regulation was further supported by the observations that levels of EREG were positively correlated with that of IGF2BP2 in clinical specimens of HNSC (**Figure 3F**) and OSCC (**Figure 3G**). In addition, a similar effect of EREG on OSCC progression was seen as manipulation of EREG expression consistently influenced the migration and invasion responses in OSCC cells (**Figure 3H-I**). These results highlight a role of IGF2BP2-regulated EREG expression in controlling OSCC invasion.

### EREG acts as a downstream regulator of IGF2BP2-mediated OSCC invasion

**Figure 4:**
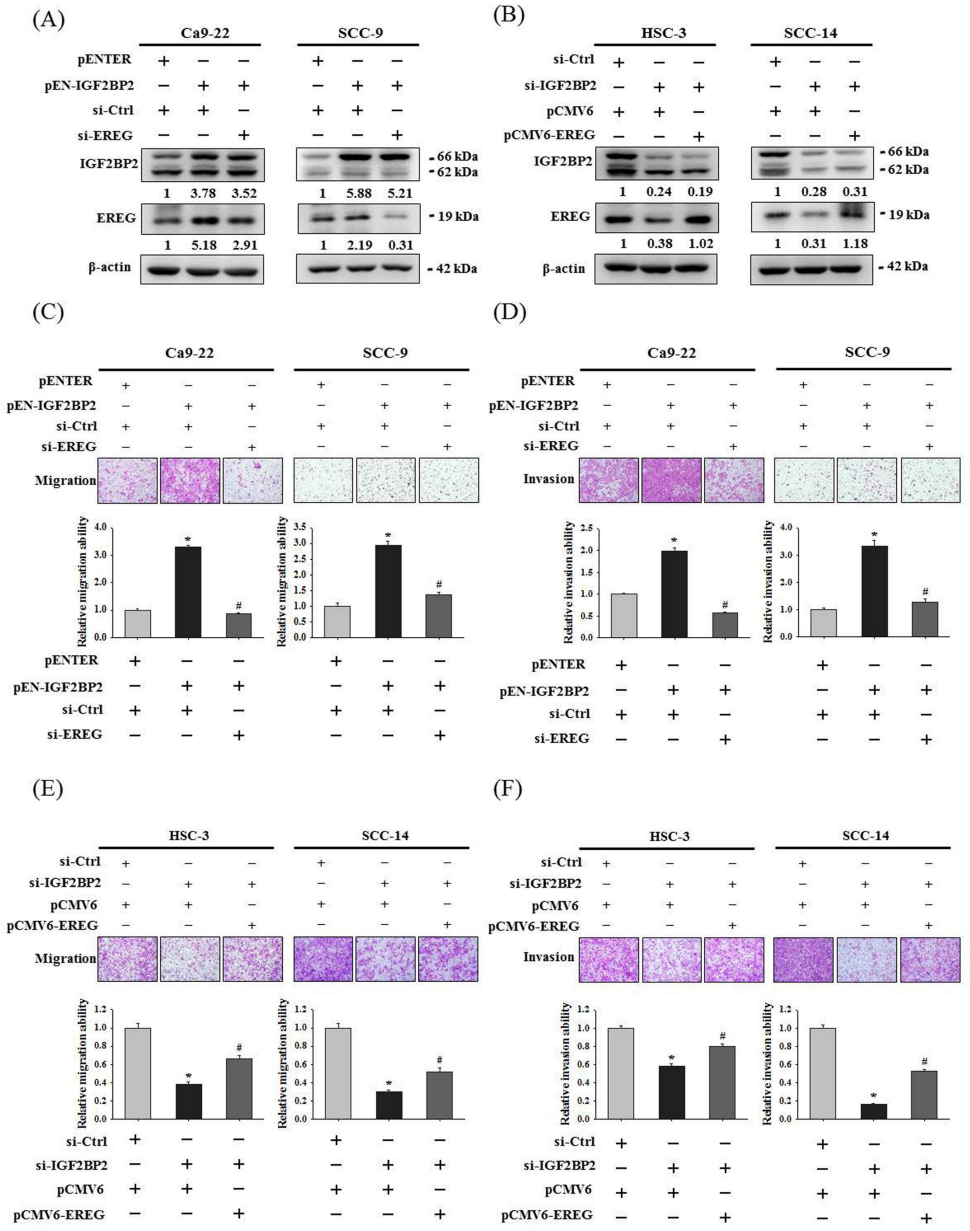
** EREG acts as a downstream regulator of IGF2BP2-mediated OSCC invasion. (A-B)** OSCC cells were co-transfected with indicated plasmid DNAs and siRNAs and evaluated for the levels of IGF2BP2 and EREG by immunoblotting. Densitometric analyses were quantified and normalized with internal controls (β-actin). **(C-F)** OSCC cells with manipulation of IGF2BP2 and EREG levels were evaluated for their migration and invasion responses in a modified Boyden chamber without and with Matrigel coating, respectively. Quantitative results are shown below. *p < 0.05, compared with the vehicle control; #*p <* 0.05, compared with IGF2BP2-overexpressing or -depleted cells, using Student's t-test.

To clarify a direct functional connection, we simultaneously manipulated IGF2BP2 and EREG in OSCC cells, including knockdown of EREG in IGF2BP2-overexpressing cells and forced expression of EREG in IGF2BP2-depleted cells, and assessed the resulting invasion responses. We found that knockdown of EREG in IGF2BP2-overexpressing Ca9-22 and SCC-9 cells dampened the induction of migration and invasion responses by IGF2BP2 (**Figure 4A, 4C, and 4D**). Reciprocally, forced expression of EREG in IGF2BP2-depleted HSC-3 and SCC-14 cells significantly restored the cell migration and invasion (**Figure 4B, 4E, and 4F**). These findings suggested that EREG acts as a potential downstream target of IGF2BP2 to modulate the invasiveness of OSCC.

### IGF2BP2-regulated EREG expression promotes epithelial-mesenchymal transition (EMT) in OSCC

**Figure 5:**
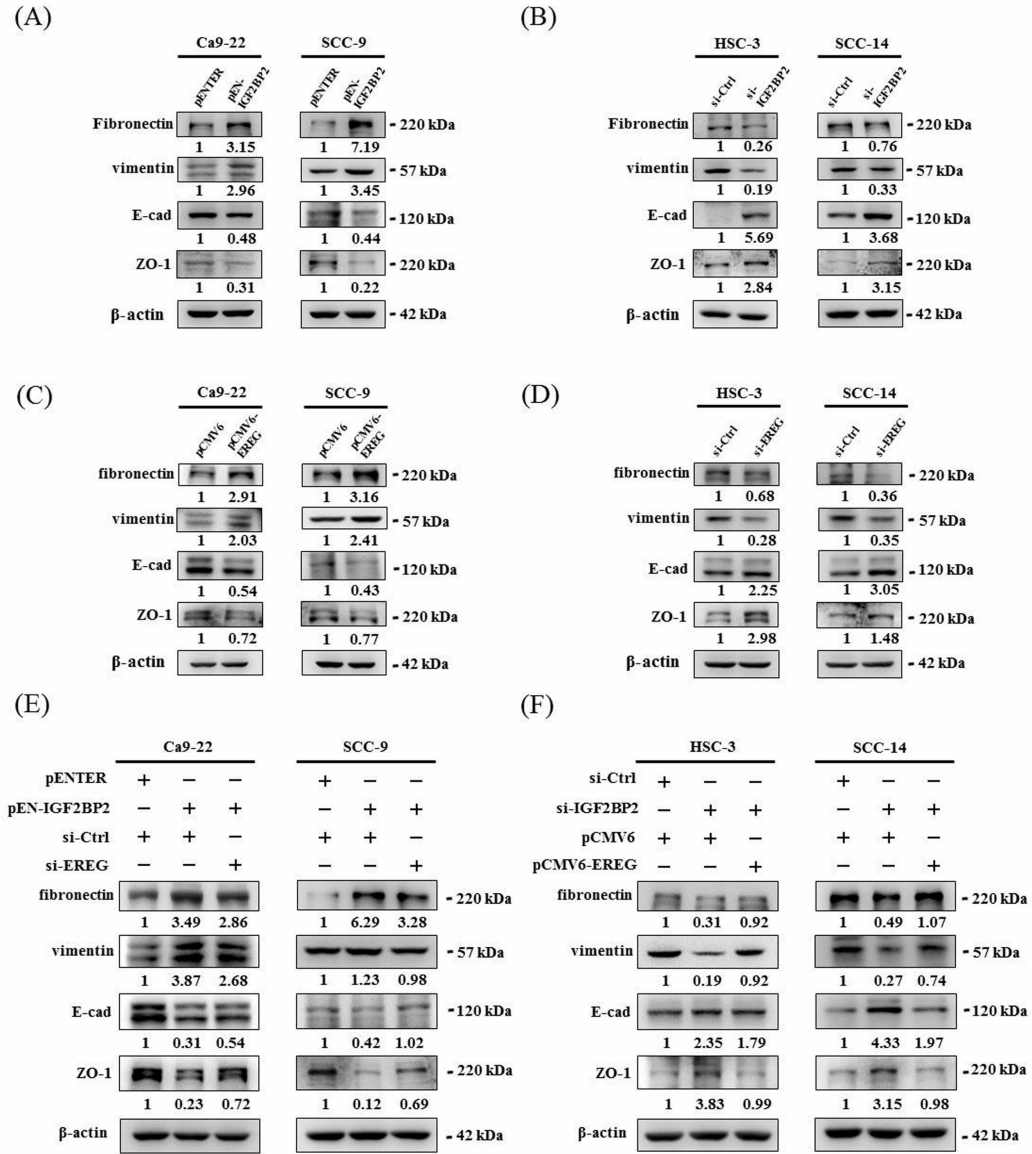
** IGF2BP2-regulated EREG expression triggers EMT in OSCC. (A-D)** OSCC cells transfected with indicated expression vectors or siRNAs were analyzed for the levels of EMT markers by immunoblotting. **(E-F)** OSCC cells with manipulation of IGF2BP2 and EREG levels by co-transfection of indicated plasmid DNAs and siRNAs were examined for the expression of EMT markers Densitometric analyses were quantified and normalized with internal controls (β-actin).

Epithelial-mesenchymal transition (EMT), involving a dramatic reorganization of the actin cytoskeleton and the concomitant switch of membrane receptors, is a process that allows cancer cells to change their adhesive repertoire and to gain migratory and invasive properties 32. We next tested whether EMT is involved in IGF2BP2-mediated OSCC invasion by assessing the levels of EMT markers. Our results reveal that forced expression of IGF2BP2 in OSCC cells enhanced the levels of mesenchymal markers (fibronectin and vimentin) and lowered that of epithelial markers (E-cadherin and ZO-1) (**Figure 5A**). On the contrary, a reduction in fibronectin and vimentin expression and an elevation in E-cadherin and ZO-1 levels were observed in IGF2BP2-depleted cells (**Figure 5B**). Similar to the effect of IGF2BP2, a promotive role of EREG levels in EMT was also detected (**Figure 5C-D**). Furthermore, knockdown of EREG weakened the promotion of EMT by IGF2BP2 (**Figure 5E**), and replenishment of EREG favored the EMT in IGF2BP2-depleted cells (**Figure 5F**). These results point out an impact of IGF2BP2-regulated EREG expression on promoting EMT in oral cancer.

### Src contributes to IGF2BP2-mediated induction of EREG expression and cell invasion in OSCC

**Figure 6:**
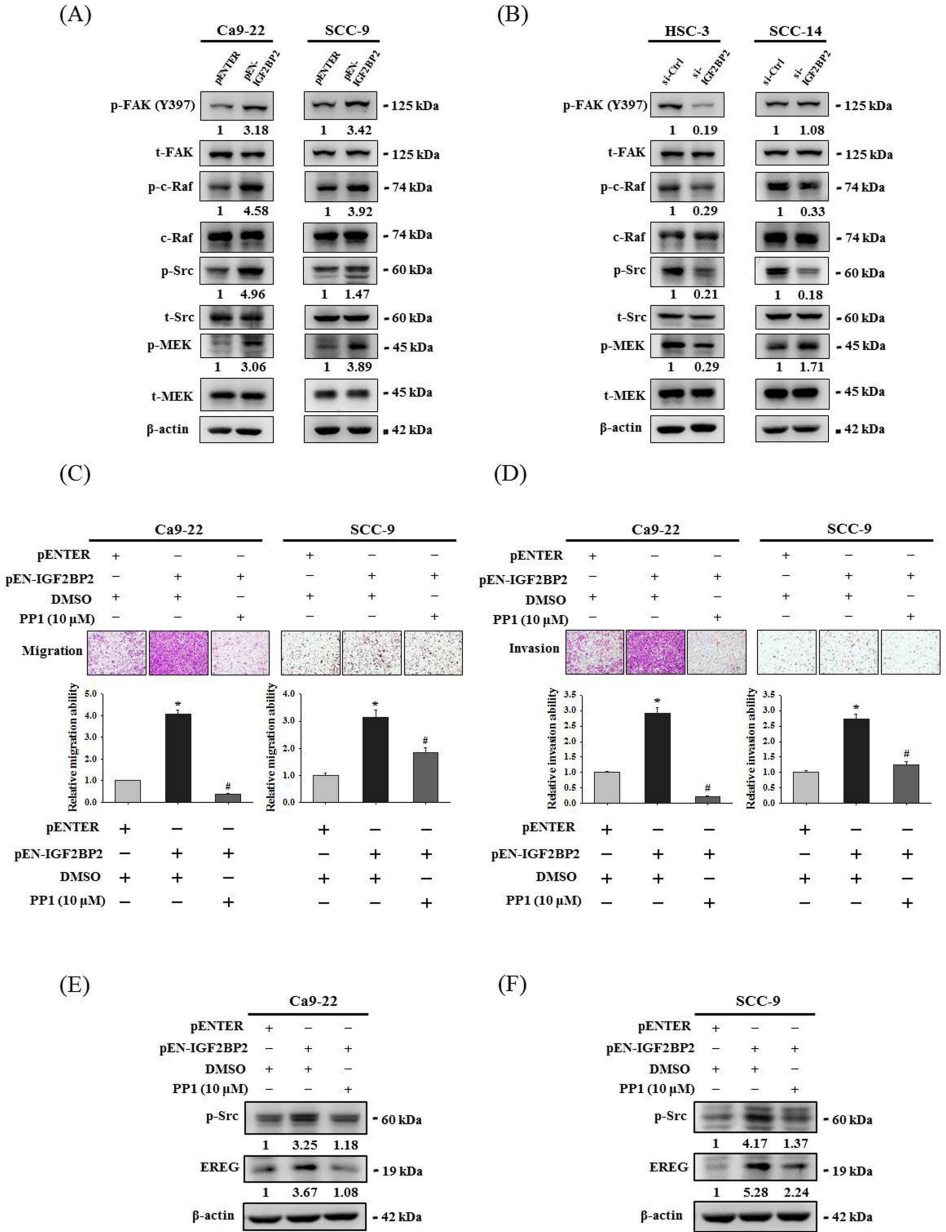
**IGF2BP2-regulated EREG expression and cell invasion are dependent on the activation of FAK/Src signaling. (A-B)** Cells were transfected with indicated plasmid DNAs **(A)** or siRNAs **(B),** and cell lysates were subjected to immunoblotting for analyzing the phosphorylation of FAK, c-Raf, Src, and MEK. Densitometric analyses of kinase activation were conducted by measuring the phosphorylated form over total levels of individual kinases. **(C-D)** At 48 hr post-transfection, Ca9-22 and SCC-9 cells were pre-treated with PP1 for 1 hr and assessed cell migration and invasion. Quantitative results are shown below. *p < 0.05, compared with the vehicle control; #*p <* 0.05, compared with IGF2BP2-overexpressing cells, using Student's t-test. **(E-F)** At 48 hr post-transfection, Ca9-22 **(E)** and SCC-9 cells **(F)** were pre-treated with PP1 for additional 6 hr and assessed for EREG expression. Densitometric analyses were quantified and normalized with internal controls (β-actin).

Focal adhesion kinase (FAK)/Src pathway represents a signaling cascade that primarily regulates cytoskeletal rearrangement and cell migration 33. Next, we evaluated the link of FAK/Src signaling with the effect of IGF2BP2 on OSCC invasion. Through examining the activation profile of FAK/Src signaling, we found that phosphorylation levels of FAK, Raf, Src, and MEK were positively regulated by IGF2BP2 expression in OSCC cells (**Figure 6A-B**). Further, we demonstrated that pharmacological blockage of Src by the treatment of PP1 in IGF2BP2-overexpressing Ca9-22 and SCC-9 cells significantly impaired the induction of cell invasion (**Figure 6C-D**) and EREG expression (**Figure 6E-F**) by IGF2BP2. These results indicate a functional involvement of Src in IGF2BP2-regulated EREG expression and OSCC invasion, providing additional insight into the mechanisms underlying the promotive role of IGF2BP2 in OSCC progression.

## Discussion

**Figure 7:**
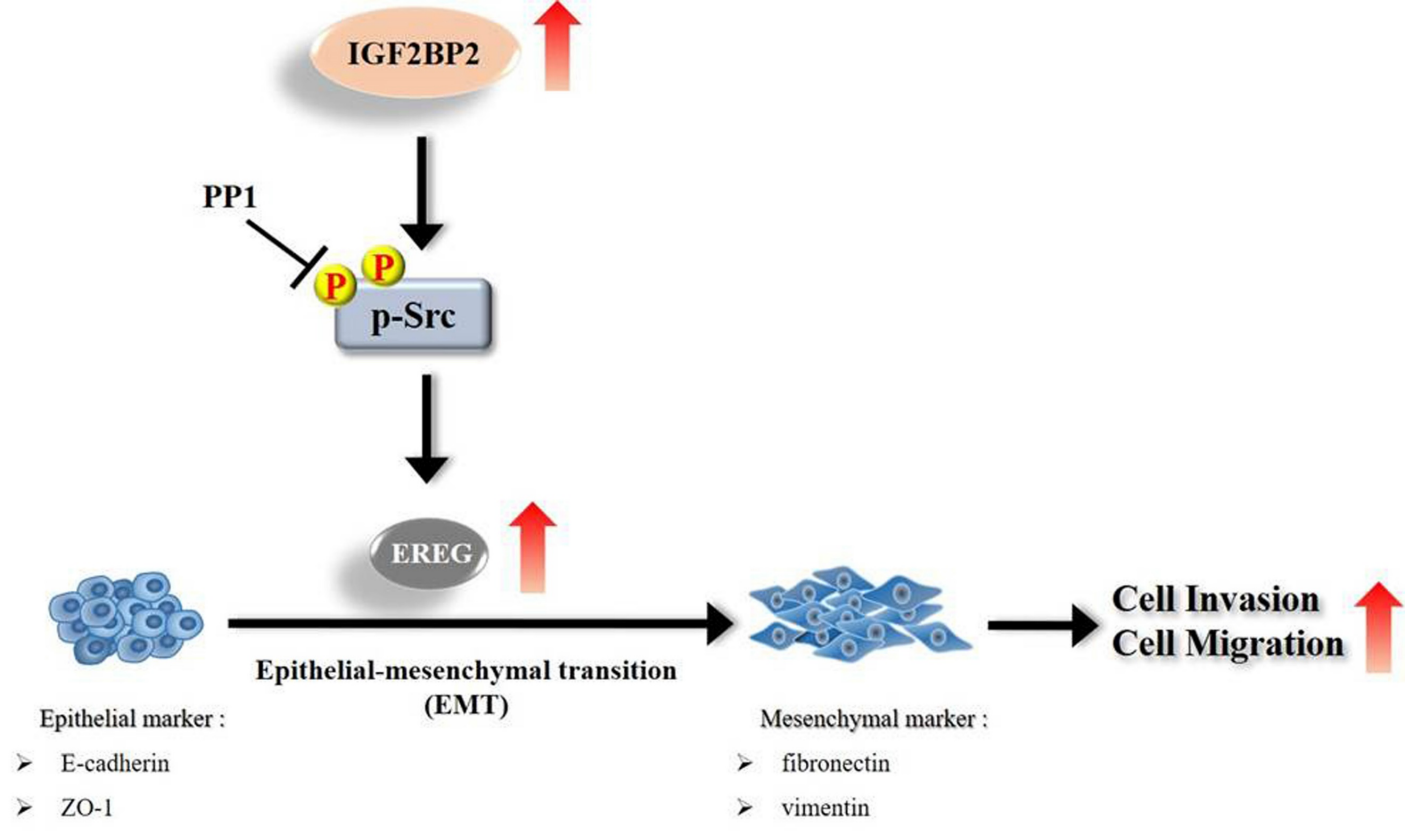
Schematic diagram of IGF2BP2-regulated gene, EREG, in modulation of oral cancer migration.

Even though current therapeutic strategies of OSCC have achieved favorable outcomes for cases with early-stage diseases, the prognosis and high mortality of patients with advanced-stage tumors remain an enormous burden. Therefore, extra treatment options seem to be needed for fighting this devastating malignancy. In this study, we provided evidence that IGF2BP2, a RNA-binding protein known to regulate the localization, stability, and translation of various RNA transcripts, elicited a promotive effect on the invasion responses of OSCC, involving the induction of EREG levels and EMT (**Figure 7**). Further investigations revealed that EREG functioned as a modulator of OSCC invasion downstream of IGF2BP2 in a FAK/Src-mediated manner. As small molecules targeting IGF2BP2 for cancer therapy have been developed 34, our results may offer potential avenues for the management of oral cancer.

IGF2BP2 has been proposed as a tumor promoter and shown to affect many cancer hallmarks through positive or negative regulation of its target genes. A recent study demonstrated that IGF2BP2 promoted the degradation of the RNA transcripts of the ATP6V1A gene, thereby impairing lysosomal function and resulting in a unique secretome that greatly enhances breast cancer cell invasiveness 20. In addition to downregulation of target genes by increased RNA degradation, IGF2BP2 was shown to interact with N6-methyladenosine (m6A)-modified RNAs and enhance the stability of these RNAs. Modification of m6A on RNAs a dynamic and reversible reaction, and dysregulation of m6A has been linked to the development and progression of cancer 35. In OSCC, IGF2BP2 has been reported to leverage the expression of an autophagy-related gene, RB1CC1 (RB1 Inducible Coiled-Coil 1), subsequently affecting cancer progression in a m6A-mediated manner 36. Moreover, IGF2BP2 promoted apoptosis of hypopharyngeal cancer by facilitating mRNA stability of the TLR2 (toll-like receptor 2) gene 37. Through positive regulation of a glycolysis-associated gene, HK2 (hexokinase 2), IGF2BP2 has been documented to control tumor metabolism in colon cancer 38. Via this m6A-dependent mechanism, upregulation of CCAR1 (Cell Division Cycle And Apoptosis Regulator 1) 39, SMAD3 (Mothers against decapentaplegic homolog 3) 40, and SNAIL2 (Snail family transcriptional repressor 2) 41 by IGF2BP2 was demonstrated to promote metastatic responses of prostate, gastric, and head and neck cancer, respectively. In this study, although an involvement of m6A was undefined, we showed a promotive role of IGF2BP2 in OSCC invasion and EMT, where EREG was identified as a downstream mediator positively regulated by IGF2BP2. Of note, accompanied with upregulation of ETV-1 (ETS-translocation variant 1), a similar effect of IGF2BP2 on orchestrating EMT and cell invasion in pancreatic cancer was observed 42. Our findings reveal that EREG represents a novel member of IGF2BP2-regulated genes in modulation of OSCC progression.

Activation of EREG signaling is central to cancer cell proliferation, metastasis, and angiogenesis 43. In oral cancer, an elevation of EREG expression was detected and associated with cancer cell invasion 44, 45. It is known that proteolytical cleavage by a disintegrin and a metalloprotease 17 (ADAM17) released soluble forms of EREG to bind and activate ERBB1/EGFR and ERBB4/Her4, ultimately functioning as a driver of metastasis 46, 47. Yet, a previous investigation indicated that mistrafficking of EREG to the apical surface of epithelial cells resulted in large-size, hyperproliferative, and locally invasive tumors, which may be related to sustained EGFR signaling by apical EREG 48. Unlike other EGFR ligands, EREG could mimic EGFR mutants by sustaining the activation of the EGFR-Erk pathway in head and neck cancers 49. Our results provided here connect the EREG/EGFR pathway to the actions of IGF2BP2 on regulation of OSCC invasion.

Our results unveil a tumor-promotive role of IGF2BP2 in OSCC progression through the induction of EREG expression and cancer cell invasion. However, additional efforts are needed to address several study limitations. One concern is that, although we demonstrated promotion of OSCC migration and invasion by manipulation of IGF2BP2 levels in *in vitro* experiments, the effects of this RNA-binding protein on oral cancer progression may be different within complex tumor microenvironments in *in vivo* settings. Extra animal experiments required to verify the oncogenic properties of IGF2BP2 in combating oral carcinogenesis. Another issue is that IGF2BP2 regulates the expression levels of target genes via m6A-dependent or -independent mechanisms. An involvement of m6A in IGF2NP2-regulated EREG expression requires further investigations.

In conclusion, we demonstrated that IGF2BP2 triggered the invasion responses of OSCC, involving the induction of EREG levels and EMT. Our data provide new mechanistic insights into the tumor-promotive features of IGF2BP2, on the management of patients with oral cancer.
